# Tetrabromobisphenol A (TBBPA)-stimulated reactive oxygen species (ROS) production in cell-free model using the 2′,7′-dichlorodihydrofluorescein diacetate (H_2_DCFDA) assay—limitations of method

**DOI:** 10.1007/s11356-016-6450-6

**Published:** 2016-03-15

**Authors:** Konrad A. Szychowski, Kamila Rybczyńska-Tkaczyk, Marcin L. Leja, Anna K. Wójtowicz, Jan Gmiński

**Affiliations:** Department of Public Health, Dietetics and Lifestyle Disorders, University of Information Technology and Management in Rzeszow, Sucharskiego 2, 35-225 Rzeszow, Poland; Department of Environmental Microbiology, Laboratory of Mycology, University of Life Sciences, Leszczyńskiego 7, 20-069 Lublin, Poland; Department of Animal Biotechnology, Animal Sciences Faculty, University of Agriculture, Redzina 1B, 30-248 Krakow, Poland

**Keywords:** TBBPA, Free radical, H_2_DCFDA, DPPH, ROS

## Abstract

Tetrabromobisphenol A (TBBPA) is a widely used brominated flame retardant, applied in a variety of commercial and household products, mainly electronic ones. Since the production of reactive oxygen species (ROS) is considered one of the principal cytotoxicity mechanisms, numerous studies undertake that aspect of TBBPA’s mechanism of action. The present study verifies if the fluorogenic substrate 2′,7′-dichlorodihydrofluorescein diacetate (H_2_DCFDA) should be used to detect ROS production induced by TBBPA. To determine the ability of TBBPA alone to stimulate the conversion of H_2_DCFDA to its fluorescent product 2’,7’-dichlorofluorescein (DCF), we used a cell-free model. In the experiments we check different cultured media also in combination with free radical scavenger N-acetyl-l-cysteine (NAC). Additionally, experiments with stable free radical 2,2-diphenyl-1-picrylhydrazyl (DPPH·) have been made. The presented data showed that TBBPA in all tested concentrations interacts with H_2_DCFDA in phosphate-buffered saline (PBS) buffer while in micromolar concentrations in the DMEM/F12 medium with and without serum. The addition of NAC inhibited the interaction of TBBPA with H_2_DCFDA. Experiments with DPPH· showed that, in the presence of NAC, TBBPA acts like a free radical. TBBPA has similar properties to free radical and is susceptible to free radical scavenging properties of NAC. Our results indicated that H2DCFDA assay cannot be used to evaluate cellular ROS production in TBBPA studies. The study connected with TBBPA-stimulated ROS production in cell culture models using the H2DCFDA assay should be revised using a different method. However, due to the free radical-like nature of TBBPA, it can be very difficult. Therefore, further investigation of the nature of TBBPA as a compound with similar properties to free radical is required.

## Introduction

Tetrabromobisphenol A (2,2-bis(4-hydroxy-3,5-dibromophenyl)propane; TBBPA) is a widely used brominated flame retardant (BFR). Currently, approximately 75 different commercial BFRs are known, from which the most common used is TBBPA. The annual demand of TBBPA was about 130,000 t in 2002, approximately 85 % of which are used in East Asia (Lyche et al. 2015). TBBPA is applied in a variety of commercial and household products, such as plastics, textiles, and electronic appliances, including computers and televisions (de Wit et al. 2010). Sellström and Jansson (1995) show that TBBPA can leak from different products and, due to its lipophilicity and environmental stability, can accumulate in living organisms (Hakk and Letcher 2003). Due to the accumulation of TBBPA in different tissue, it is necessary to verify the toxicity mechanism of this compound. One of the well-known principal mechanisms of cytotoxicity leading to cell death is the production of reactive oxygen species (ROS). ROS includes oxygen radicals (superoxide and hydroxyl) as well as some non-radical derivatives of molecular oxygen (O_2_) such as hydrogen peroxide (H_2_O_2_) (Halliwell 1999). Furthermore, ROS is only one type of a wide group of free radicals, which can be defined as reactive chemical species having a single unpaired electron in an outer orbit (Riley 1994; Liochev 2013). It is well known that bromine is a very reactive element, which reacts with many substrates in the form of bromine radical (Winterbourn 2008; Halliwell 2015). This is also the reason why bromine does not exist as a free element in nature. The effectiveness of BFR lies in its ability to release active bromine atoms (called low-energy free radicals) into the gas phase before the material reaches its ignition temperature (Troitzsch 1998). The most popular method used to measure the level of cellular ROS formation is 2′,7′-dichlorodihydrofluorescein diacetate (H_2_DCFDA) assay. So far, it has been shown that TBBPA can increase ROS production in different cell culture models and, in that way, cause apoptosis (Reistad et al. 2005, 2007; Al-Mousa and Michelangeli 2012; Hendriks et al. 2012, 2014; Ziemińska et al. 2012). In the abovementioned studies the H_2_DCFDA assay was applied. However, the authors did not check the ability of TBBPA alone to react with H_2_DCFDA. The recently published data using the H_2_DCFDA assay showed that TBBPA stimulated ROS production not only in cell culture model but also in cell-free models (Tetz et al. 2013; Szychowski and Wójtowicz 2016). These data suggests that not only the H_2_DCFDA assay is inadequate to measure ROS formation, but it also creates new questions about the nature of the compound. For this reason, the aim of the present study was to assess if TBBPA on its own has free radical properties.

## Materials and methods

### Reagents

TBBPA, H_2_DCFDA, phosphate-buffered saline (PBS) without Ca^2+^ and Mg^2+^, 2,2-diphenyl-1-picrylhydrazyl (DPPH·), fetal bovine serum (FBS), hydrogen peroxide (H_2_O_2_), ethanol, and dimethyl sulfoxide (DMSO) were purchased from Sigma-Aldrich (St. Louis, MO, USA). The DMEM/F12 medium was purchased from ATCC (Manassas, VA, USA). H_2_DCFDA stock solution was prepared by dissolving the compounds in DMSO. TBBPA, NAC, and DPPH· were dissolved in ethanol.

### Measurement of TBBPA-stimulated fluorescence

The fluorogenic dye H_2_DCFDA was used to detect ROS production. Normally, after diffusion into the cell, H_2_DCFDA is deacetylated by cellular esterases into a non-fluorescent compound that is subsequently oxidized by ROS into 2′,7′-dichlorofluorescein (DCF) (Gomes et al. 2005).

To determine the ability of TBBPA alone to stimulate the conversion of H_2_DCFDA to its fluorescent product DCF, we used a cell-free model. Dilution of 5 μM H_2_DCFDA and increasing concentrations of TBBPA (0.1–100 μM) were added to 96-well plates containing PBS buffer without Ca^2+^ and Mg^2+^ or serum-free DMEM/F12 or DMEM/F12 supplemented with 5 % FBS in the final volume of 100 μL. The fluorescence was measured 30 and 60 min after the addition of TBBPA according to a protocol previously described by Szychowski and Wójtowicz (2016). The same experiment was performed with the presence of 10 μM *N*-acetyl-l-cysteine (NAC) ROS scavenger. Medium with 0.3 % hydrogen peroxide (H_2_O_2_) was used as a positive control. DCF fluorescence was detected using a microplate reader (FilterMax F5) at maximum excitation and emission spectra of 485 and 535 nm, respectively. The data was analyzed using Multi-Mode Analysis software and was normalized to the fluorescence in a vehicle-treated control (% of control).

### Measurement of DPPH· free radical scavenging assay

2,2-Diphenyl-1-picrylhydrazyl (DPPH·) is a stable free radical molecule. DPPH· has an absorption band at 515 nm which disappears upon reduction by an antiradical compound. DPPH· is applied in laboratory research as a monitor of chemical reactions involving radicals, most notably as a common antioxidant assay (Sharma and Bhat 2009).

DPPH· free radical scavenging was analyzed according to Brand-Williams et al. (1995). In the first experiment, increasing concentrations of TBBPA were mixed with 100 μL of 250-μM DPPH· (≈0.01 % approximately) solution in 96 % ethanol to final concentrations of 0.1, 1, 10, 50, and 100 μM of TBBPA. In the second experiment, increasing concentrations of TBBPA solutions and 1 μM of NAC were mixed with 100 μL of 250 μM DPPH· solution in 96 % ethanol to final concentrations of 0.1, 1, 10, 50, and 100 μM of TBBPA. Following 30 min of incubation at room temperature, the absorbance of the sample was measured at *λ* = 515 nm using 96 % ethanol as a blank sample. DPPH· absorbance was detected using a microplate reader (FilterMax F5), and the data was analyzed using Multi-Mode Analysis software and was normalized as described above (% of control). Due to its ROS scavenging properties, NAC was used as a positive control. The percentage of DPPH· scavenging was calculated for each sample based on the equation:

$$ \%\ \mathrm{of}\ {\mathrm{DPPH}}^{\bullet}\mathrm{s}\mathrm{c}\mathrm{avenging} = \left[1\ \hbox{--}\ \left(\mathrm{A}\mathrm{s}/\mathrm{A}\mathrm{c}\right)\right]\times 100\% $$

where As—absorbance of the sample and Ac—absorbance of the control sample (DPPH· solution).

### Statistical analysis

The data was presented as the means ± SEM of four independent experiments. Each treatment was repeated eight times (*n* = 8) and measured in quadruplicate. The average of the quadruplicate samples was used for the statistical analyses. Statistical analysis was performed on the original results. Considering the different data from the measurement of fluorescence or absorbance, the results were presented as percentage of controls. The data were analyzed via one-way analysis of variance (ANOVA) followed by Tukey’s multiple comparison procedure in STATISTICA 10 software. *p < 0.05, **p < 0.01, and ***p < 0.001 vs. the control.

## Results

### Effect of TBBPA on H_2_DCFDA fluorescence in cell-free conditions

**Fig. 1:**
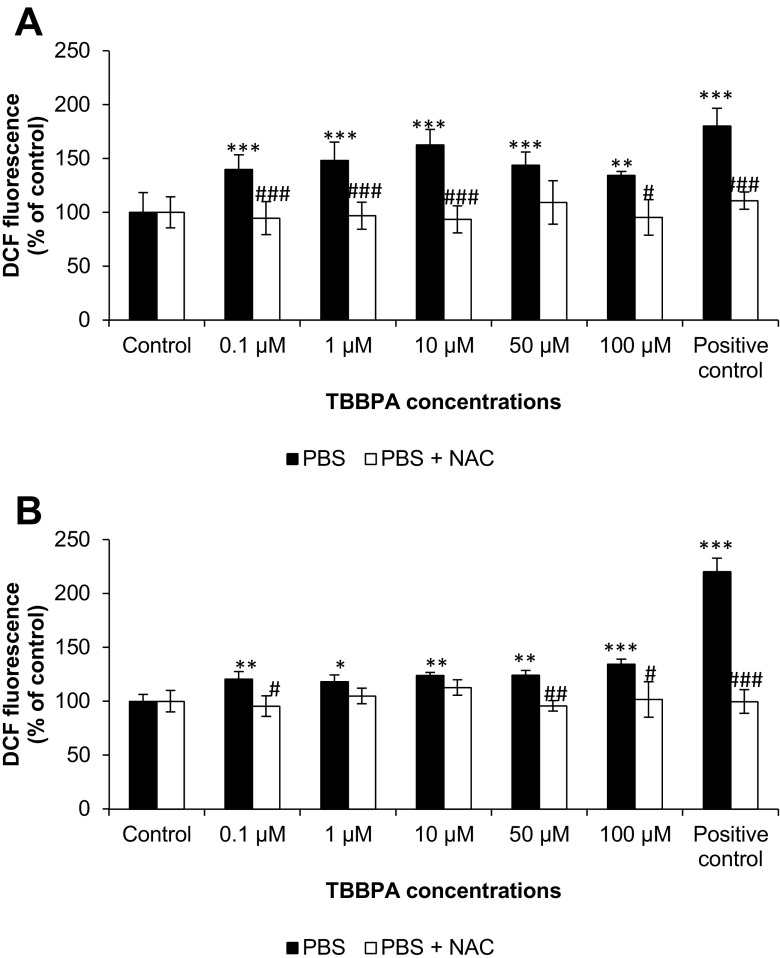
The effect of increasing concentrations of TBBPA (0.1, 1, 10, 50, and 100 μM) on the DCF fluorescence in cell-free PBS without Ca^2^+ and Mg^2^+ buffer and with addition of NAC after 30 min (**A**) and 60 min (**B**). Medium with 0.3 % hydrogen peroxide (H_2_O_2_) was used as a positive control. The data is expressed as the means ± SEM of four independent experiments, each of which consisted of eight replicates per treatment group. *p < 0.05, **p < 0.01, and ***p < 0.001 vs. the control. #*p* < 0.05, ##*p* < 0.01, and ###*p* < 0.001 vs. the group without NAC ROS scavenger

DCF fluorescence increased significantly in all tested concentrations of TBBPA (0.1, 1, 10, 50, and 100 μM) in PBS buffer without Ca^2+^ and Mg^2+^ after 30 min (increase compared to control by 40.03, 48.40, 62.72, 43.74, and 34.32 %, respectively) (Fig. 1a). After 60 min, fluorescence was continued. However, it was weaker than in the previous time period (increase compared to control by 20.59, 18.39, 24.11, 24.25, and 34.65 %, respectively) (Fig. 1b). The addition of NAC reduced the fluorescence caused by TBBPA in the whole time period.

**Fig. 2:**
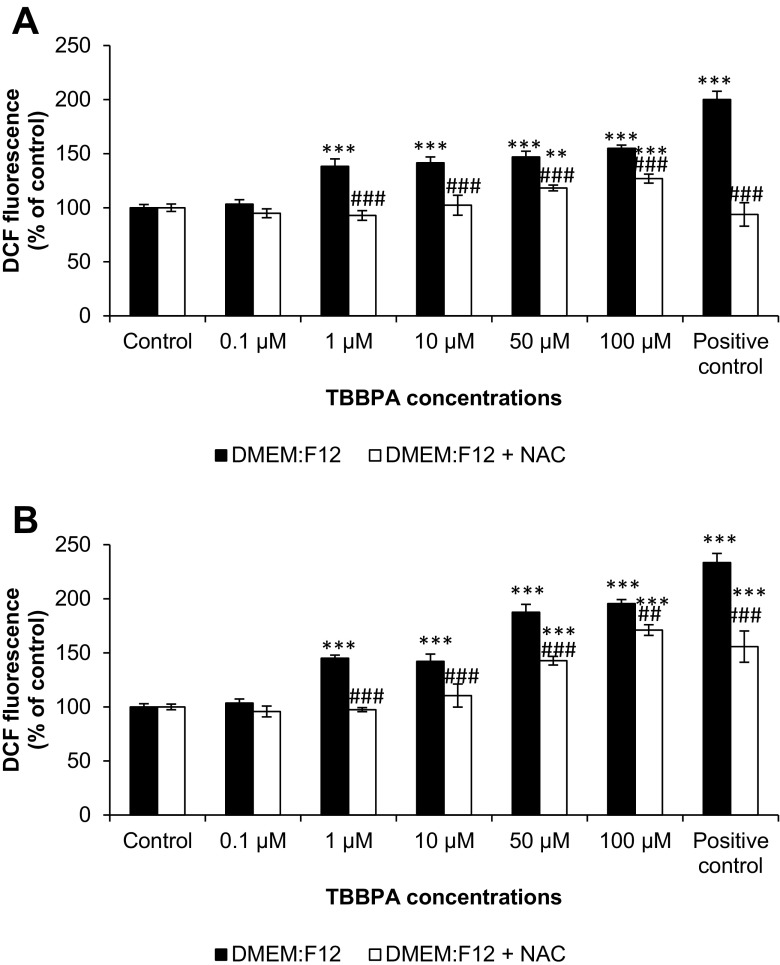
The effect of increasing concentrations of TBBPA (0.1, 1, 10, 50, and 100 μM) on the DCF fluorescence in cell-free DMEM/F12 medium and with addition of NAC after 30 min (**A**) and 60 min (**B**). Medium with 0.3 % hydrogen peroxide (H_2_O_2_) was used as a positive control. The data is expressed as the means ± SEM of four independent experiments, each of which consisted of eight replicates per treatment group. **p < 0.01 and ***p < 0.001 vs. the control. ##*p* < 0.01 and ###*p* < 0.001 vs. the group without NAC ROS scavenger

In the DMEM/F12 medium, DCF fluorescence increased starting from 1- to 100-μM concentrations of TBBPA after 30 min (increase compared to control by 38.25, 41.55, 46.96, and 54.86 %, respectively). The addition of NAC reduced the fluorescence caused by TBBPA to the control level in 1 and 10 μM of TBBPA. Partial reduction of fluorescence to 118.28 and 126.91 % was observed in 50- and 100-μM TBBPA concentrations, respectively (Fig. 2a). After 60 min, fluorescence was intensified in 1 to 100 μM of TBBPA (increase compared to control by 44.99, 42.09, 87.64, and 95.56 %, respectively). The addition of NAC reduced fluorescence caused by TBBPA to the control level in 1 and 10 μM of TBBPA. Partial reduction of fluorescence to 142.75 and 171.10 % was observed in 50- and 100-μM TBBPA concentrations, respectively (Fig. 2b).

**Fig. 3:**
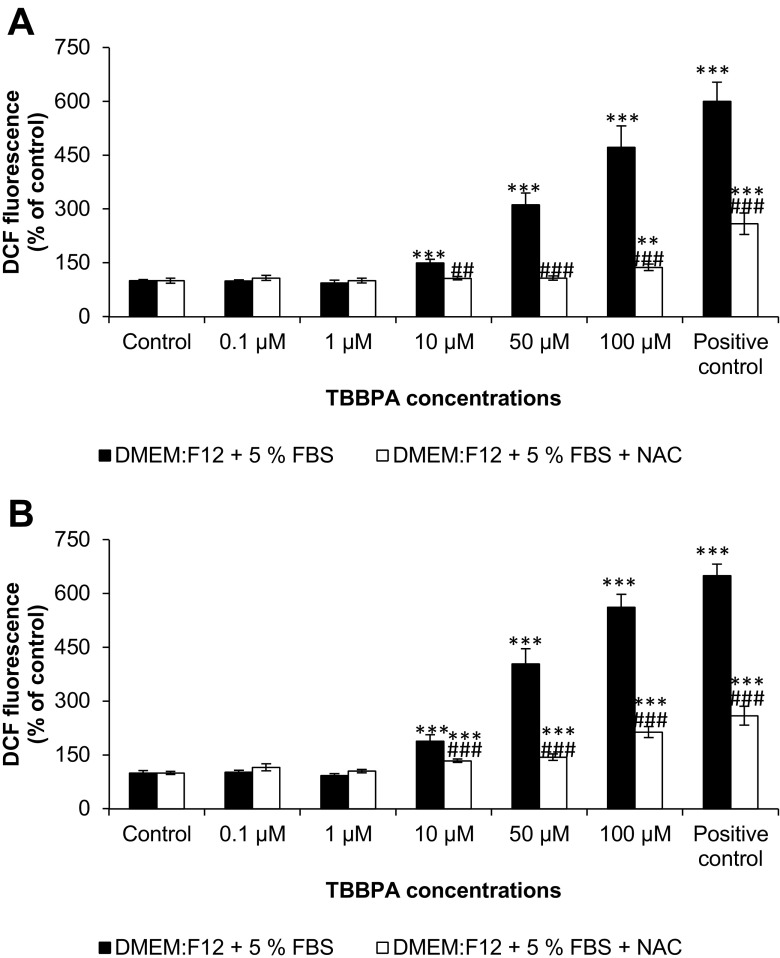
The effect of increasing concentrations of TBBPA (0.1, 1, 10, 50, and 100 μM) on the DCF fluorescence in cell-free DMEM/F12 medium supplemented with 5 % of FBS and with addition of NAC after 30 min (**A**) and 60 min (**B**). Medium with 0.3 % hydrogen peroxide (H_2_O_2_) was used as a positive control. The data is expressed as the means ± SEM of four independent experiments, each of which consisted of eight replicates per treatment group. **p < 0.01 and ***p < 0.001 vs. the control. ##*p* < 0.01 and ###*p* < 0.001 vs. the group without NAC ROS scavenger

In the DMEM/F12 medium supplemented with 5 % FBS DCF fluorescence increased starting from 10- to 100-μM concentration of TBBPA after 30 min (increase compared to control by 50.06, 211.39, and 371.79 %, respectively). The addition of NAC reduced the fluorescence caused by TBBPA to the control level in 10 and 50 μM of TBBPA, and partial reduction of fluorescence to 137.09 % was observed in 100-μM concentration of TBBPA (Fig. 3a). After 60 min, fluorescence was intensified (increase compared to control by 88.85, 303.95, and 462.41 %, respectively). The addition of NAC reduced the fluorescence caused by TBBPA in 10, 50, and 100 μM of TBBPA to 133.91, 144.02, and 213.73 %, respectively (Fig. 3b).

### Measurement of DPPH· free radical scavenging activity

**Fig. 4:**
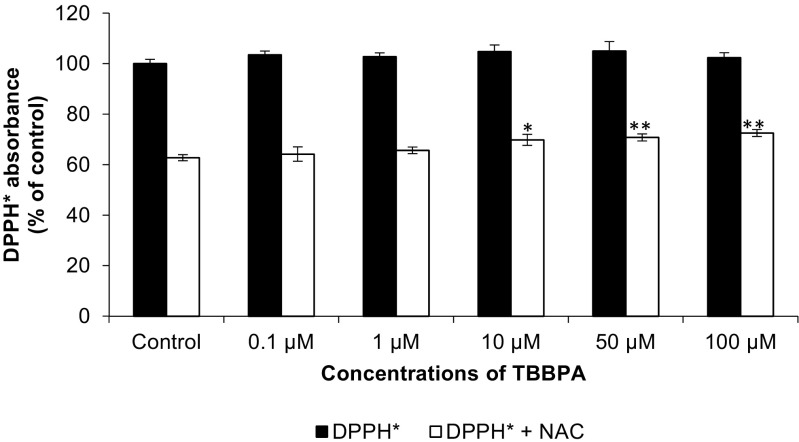
The effect of increasing concentrations of TBBPA (0.1, 1, 10, 50, and 100 μM) on the DPPH· free radical and NAC ability of free radical scavenging. The data is expressed as the means ± SEM of four independent experiments, each of which consisted of eight replicates per treatment group. *p < 0.05 and **p < 0.01 vs. the control with NAC

The free radical nature of TBBPA was evaluated using DPPH· stable free radical scavenging assays. The reaction is based on decolorization of free radical solution following incubation with analyzed substrates. The decrease of absorption after adding substrates is directly proportional to the number of DPPH· radicals (Alam et al. 2013). Our data demonstrated that after 30 min, DPPH· absorbance was not changed in any of the tested concentrations of TBBPA. In an experiment with NAC, 10, 50, and 100 μM of TBBPA increase DPPH· absorbance compared to DPPH· with NAC control by 7.04, 8.01, and 9.77 %, respectively (Fig. 4).

## Discussion

Our study examined for the first time the impact of TBBPA on H_2_DCFDA fluorescence without cells in PBS buffer, DMEM/F12, and DMEM/F12 with 5 % of FBS media. The obtained results showed that TBBPA in all tested concentrations interacted with H_2_DCFDA in PBS buffer and caused a significant increase in fluorescence. The reaction was diminished by the addition of the free radical scavenger *N*-acetyl-l-cysteine (NAC). Our data shows that in cell-free PBS buffer model TBBPA acts like a free radical molecule. Similar results have been obtained in DMEM/F12 and DMEM/F12 with 5 % of FBS. In the DMEM/F12 medium, the increase in fluorescence started from 1 μM of TBBPA, but in the DMEM/F12 medium supplemented with 5 % FBS, it started from 10 μM of TBBPA. In both studied variants of culture media supplemented with NAC, only partial reduction of fluorescence stimulated by TBBPA was observed. So far, only two papers have shown that TBBPA interacts with H_2_DCFDA without the cells in serum-free neurobasal medium and serum-free HBSS solutions (Tetz et al. 2013; Szychowski and Wójtowicz 2016). However, the use for the first time of NAC free radical scavenger provides new evidence on TBBPA’s free radical nature.

It is known that H_2_DCFDA assay is sensitive to many potential compounds such as medium components, serum, heme, heme proteins, metalloporphyrins and bovine serum albumin, and many others which have previously been revised by Chen et al. (2010). It is also known that after the penetration of the cellular membrane, H_2_DCFDA becomes deacetylated by cellular esterases into the non-fluorescent compound H_2_DCF, which is then oxidized by ROS into the fluorescent product DCF (Gomes et al. 2005). Similar to all kinds of serum, the FBS used contains extracellular esterases which explains more intense fluorescence caused by TBBPA in the DMEM/F12 medium with 5 % of FBS. Esterases present in FBS convert H_2_DCFDA to H_2_DCF, which react much easier with TBBPA and caused the much higher fluorescence than in the DMEM/F12 medium without FBS. The presence of extracellular esterases is the reason of high background fluorescence. This background fluorescence is probably responsible for less sensitivity of the H_2_DCFDA assay in DMEM/F12 with serum. In the experiments in the medium containing FBS, the fluorescence effect caused by TBBPA starts from higher concentrations compared to PBS.

The presence of free radicals causes a chain reaction leading to consecutive oxidation. Free radicals attack molecules like fat, proteins, DNA, sugar, etc., and the newly damaged molecule unfortunately becomes a new free radical which attacks a new molecule (Feychting et al. 2001). That phenomenon of free radical chain reaction can also exist in our experimental model, which may explain why NAC does not inhibit all fluorescence caused by high micromolar concentrations of TBBPA. Our data is consistent with previous studies (Tetz et al. 2013; Szychowski and Wójtowicz 2016), where the authors have demonstrated that the method is inappropriate for the measurement of cellular ROS production after TBBPA stimulation in cell culture models.

To elucidate the nature of TBBPA, DPPH· free radical scavenger assay has been made. The DPPH· radical scavenging assay is an easy, rapid, and sensitive method for the detection of antioxidants. A number of methods are available for the determination of free radical scavenging activity, but the assay employing DPPH· has received the most attention owing to its ease of use and its convenience. Our data showed that in an experiment with simultaneous addition of NAC and TBBPA to DPPH·, absorbance increased slightly as compared to control DPPH· with NAC. The data suggests that TBBPA acts similar to free radical competing with DPPH· for NAC scavenging. Due to the nature of bromine, bromophenols are more susceptible to reductive debromination rather than the oxidation process (Guo et al. 2012). As it has been mentioned, bromine is used in BRF due to its ability to be low-energy free radicals (Troitzsch 1998). Therefore, in our opinion, it is possible that bromine can also be responsible for the transformation of TBBPA in a free-like radical molecule. A similar suggestion was reported by Arbeli et al. (2006) where TBBPA was reductively dehalogenated by microorganisms and acted as an electron acceptor which is characteristic of free radicals. What is important is that because TBBPA exhibits free radical-like properties, it is also possible that it can cause more damage in cells by initiating a free radical chain reaction. Nevertheless, we suspect that it can be very difficult to detect cellular ROS production stimulated by TBBPA due to its free radical-like nature.

## Conclusion

In the light of our data, H_2_DCFDA assay cannot be used in cell culture experiments with TBBPA. TBBPA acts as a free radical-like molecule, and it is susceptible to the scavenging properties of NAC. The authors suggest that the data regarding TBBPA-stimulated ROS production in cell culture models using the H_2_DCFDA assay should be revised using a different method. However, due to the free radical-like nature of TBBPA, it can be very difficult. Therefore, further investigation of the nature of TBBPA is required.

## Abbreviations

BFR: Brominated flame retardant
DMSO: Dimethyl sulfoxide
DPPH·: 2,2-Diphenyl-1-picrylhydrazyl
FBS: Fetal bovine serum
H_2_DCFDA: 2′,7′-Dichlorodihydrofluorescein diacetate
NAC: *N*-acetyl-l-cysteine
PBS: Phosphate-buffered saline
ROS: Reactive oxygen species
TBBPA: Tetrabromobisphenol A
